# SITC 26^th^ annual meeting – summary

**DOI:** 10.1186/1479-5876-10-105

**Published:** 2012-05-23

**Authors:** Emanuela Romano, Denise Nardelli-Haefliger, Alena Donda, Stephanie Corgnac, Pedro Romero

**Affiliations:** 1 Department of Oncology, University Hospital of Lausanne, Switzerland, Switzerland; 2 Urology Service, University Hospital of Lausanne, Lausanne, Switzerland; 3 Ludwig Center for Cancer Research, University of Lausanne, Lausanne, Switzerland; 4 Ludwig Center for Cancer Research, University of Lausanne, Orthopedic Hospital, Avenue Pierre-Decker 4, 1011, Lausanne, Switzerland

## Abstract

The 26th annual meeting of the Society for Immunotherapy of Cancer took place in Bethesda on November 4 to 6, 2011 and was organized by Charles G. Drake (*Johns Hopkins University)* Dolores J. Schendel (*Helmholtz Zentrum Muenchen – German Research Center for Environmental Health Institute of Molecular Immunology)*, Jeffrey Schlom (*National Cancer Institute, National Institutes of Health), and* Jedd D. Wolchok (*Memorial Sloan-Kettering Cancer Center).* It was an event marked by a number of extraordinary circumstances: it attracted a record attendance of 805 participants from 24 different countries. The gathering came in the wake of great as well as very sad news for the tumor immunology community. Good news included the approval of anti-CTLA-4 as a therapy for metastatic melanoma in April and the announcement in early October of the Nobel Prize in Physiology and Medicine awarded to pioneering studies in the field of immunology. Indeed, one part of the prize went to Dr. Bruce Beutler, Scripps Research Institute, La Jolla, USA and Dr. Jules Hoffman, Institute for Molecular Cell Biology, Strasbourg, France, for their discoveries in innate immunity and the other part to Dr. Ralph Steinman, The Rockfeller University, New York, for his discovery of dendritic cells. Sad news was the losses of two giants in the field. Jürg Tschopp of the University of Lausanne in March and Ralph Steinman, who passed away just three days before his Nobel Prize announcement. The loss of these two charismatic scientific leaders was particularly sad for the Annual Meeting as both J. Tschopp and R. Steinman were confirmed speakers at this meeting: the former to deliver the keynote lecture and the latter as recipient of the Richard V. Smalley prize.

## Richard V. Smalley, MD memorial lectureship: Ralph M. Steinman, MD and plenary session biology and applications of dendritic cells

The first plenary session was a memorial to R. Steinman. *J. Banchereau*, Roche, NJ, a close collaborator and friend, made a vibrant recount of Dr. Steinman’s life and scientific achievements. After reporting together with Z. Cohn the new cell type and proposing the term “dendritic cell” in 1973, R. Steinman devoted the remaining of his scientific career at The Rockefeller University to study the properties of this cell. His efforts led him to what we call today translational research, taking dendritic cells (DCs) into medicine and designing therapeutic vaccines. One of Steinman’s quotes evoked by Dr. Banchereau was most fitting to the core business of the Annual Meeting: “Immunology has the potential to identify vaccines, i.e.: antigen-specific, durable, non-noxious prevention and therapies for infections, cancer, allergy, autoimmunity and, transplantation”. Then, Dr. Banchereau summarized their two approaches to DC vaccines: ex vivo loading with the target antigens and in vivo targeting of antigens to DC via the many receptors expressed at their cell surface such as MMR, DEC-205, DC-SIGN, Langerin, DCIR, LOX-1, ASGPR, CLEC-6, Dectin-1, MARCO, CD1d, CD40. Interestingly, not all receptors are equivalent and targeting DCs via distinct lectins leads to distinct types of immune responses. In his closing remarks, Banchereau called for increased collaboration between academia and pharmaceutical industry with the aim of widening the base of target identification and accelerating the discovery and transfer of new therapeutic options to patient care.

*R. Seeder* of the Vaccine Research Center, NIAID, discussed important issues on the formulation and delivery of proteins to DCs in order to optimize T cell immunity and the use of “prime-boost immunization” with protein and viral vaccines to improve T cell immunity. The use of protein – TLR agonist conjugates would be particularly advantageous to target receptors on DCs. He made the case for TLR8 agonists, as TLR8 is expressed in three major DC subsets including conventional DC, plasmacytoid DC and the recently described cross-presenting, CD141/BDCA-3, XCR1, TLR3 and TLR8 expressing DC, human counterpart of the CD8α murine DC. Testing of this idea with model antigens in mice showed that aggregation of protein-imiquidazole conjugates enhanced uptake by DC and that multiple DC subsets participated in the initiation of specific T cell immunity. He also demonstrated that specific CD8 T cells cross-primed by protein conjugates were dramatically boosted by a recombinant pox vector (NYVAC), suggesting its use as part of protein-based therapeutic vaccine regimens.

*E. Romano*, University Hospital of Lausanne, Switzerland, discussed results generated while at J. W. Young’s laboratory at Memorial Sloan-Kettering Cancer Center. She found that human Langerhans cells can stimulate robust CTL responses against tumor antigens, including WT-1. While these cells do not make significant amounts of IL-12, they express by far the highest amounts of IL-15Rα which together with IL-15 support the rapid generation of potent tumor reactive CTLs. *Z. N. Berneman*, Antwerp University Hospital, Belgium, presented promising results using DC vaccines for leukemia in an adjuvant post-remission setting. The vaccine consists of autologous monocyte derived DCs that are electroporated with mRNA encoding for WT-1, a major transcription factor that is overexpressed in acute myeloid leukemia and in a variety of solid tumors. It was administered to AML patients during remission following polychemotherapy. By measuring WT-1 mRNA in serum, it was possible to detect the conversion of partial remission into complete remission following vaccination in 2 out of 3 patients, and the induction of molecular remission following vaccination in 8 out of 17 AML patients was detected. Several immunological effects correlated with clinical outcome including increased levels of activated NK cells after vaccination and increased WT1-specific tetramer + CD8 T cells post-vaccination that correlated with long term clinical responses (CR lasting over 3 years). Vaccination with genetically engineered DCs may be an attractive strategy to prevent AML relapse and warrants a phase II study in high risk AML patients. *S-R Woo,* in the laboratory of T. F. Gajewski, University of Chicago, reported the innate immune sensing of tumors via the host STING pathway. STING, stimulator of interferon genes, was identified in 2008 as a molecule essential to for effective innate immune signaling processes. It is an endoplasmic reticulum adaptor able to activate both NF-kB and IRF3 transcription pathways to induce expression of IFN-α and IFN-β. The question addressed was the nature of the DNA sensor in DCs that drives the STING pathway in response to tumor DNA. The results suggested that the intracellular DNA sensor IFI/p204, a PYHIN protein, is required.

*N. Bhardwaj* closed the first session by discussing modulation of DC function by the tumor microenvironment. She presented a story of a complex network of interactions in the processing and presentation of peptides derived from the matrix metalloproteinase 2 (MMP2). MMP2 is recognized by both CD8 and CD4 T cells in humans. Priming of inflammatory MMP2-specific Th2 CD4 T cells required active MMP-2. Interestingly, MMP2 acts as an endogenous Th2-conditioner for other melanoma associated antigen-specific T cell responses. MMP2 induces OX40L expression and inhibits IL-12 production by DCs both of which are critical for Th2 priming. MMP2 blocks IL-12 production via a STAT1- and IFNβ/IFNAR-dependent mechanism. While the conventional receptors for MMP2 on DCs were not involved in imprinting DCs with the Th2 priming capacity, it was found that its ability to directly degrade IFNAR1 on DCs was the likely mechanism for imprinting. Finally, her work also showed that upregulation of OX40L by MMP2 occurs via its binding to TLR2 on the surface of DCs.

## Uncoupling negative regulation in the tumor microenvironment

*D. Pardoll*, MD, PhD (John Hopkins University, School of Medicine) co-chaired the session with *H. Zarour*, MD (University of Pittsburgh). *D. Pardoll* discussed on the therapeutic manipulation of the PD-1 checkpoint pathway. In tumor-infiltrating lymphocytes (TILs) a high proportion of CD8 T cells express PD-1, while neighboring tumor cells express the ligand PD-L1. This is linked to both an adaptive resistance (i.e.: IFN-γ secretion by infiltrating T cells inducing PD-L1 expression via STAT) and an inflammatory phenotype (i.e.: increased proportion of PD-L1 expression in primary melanomas compared with metastatic lesions). PD-1 blockade (MDX 1106, Brystol-Myers Squibb) led to some tumor regression associated with increased TIL CD8 T cells and correlation with positive PD-L1 expression by tumor cells. *S. Kerkar*, MD (National Cancer Institute) reported on IL-12 triggering of an inflammatory gene signature that reverses dysfunctional antigen-presentation by myeloid derived cells residing within tumors. In a B16 mouse melanoma model, transferred IL-12-expressing CD8 T cell showed enhanced anti-tumor activity through reprogramming of tumor-infiltrating myeloid-derived cells leading to efficient cross-presentation of tumor-antigen. *J. Kline*, MD (University of Chicago) showed how acute myeloid leukemia (AML) promotes immune evasion via the induction of antigen-specific T cell deletion. Comparison of AML murine models of solid and disseminated tumors showed an impaired antigen-specific T cell response in the latter. This was not linked to suppression by Treg or MDSC, but to direct induction of T cell deletion. Interestingly, CD40 ligation was able to prevent T cell dysfunction. H. Zarour, MD (University of Pittsburgh) discussed how targeting multiple inhibitory pathways may reverse melanoma-induced T cell dysfunction. NY-ESO-1-specific CD8 T cells co-expressing PD-1 and Tim-3 exhibited high degree of dysfunction, which could be restored after blockade of both inhibitors. BTLA is also upregulated in NY-ESO-1-specific T cells with half of the cells co-expressing PD-1. This is in contrast to the low expression of these inhibitory molecules on virus-specific T cells. Studies ex-vivo showed that expression of PD-1 and Tim-3, but not BTLA, correlates with levels of T cell function.

## Characterization of Inflammatory infiltrates in Human cancers

The session was co-chaired by *W. H. Fridman*, MD, PhD (INSERM, France) and *G. Coukos*, MD, PhD (University of Pennsylvania Medical Center). *G. Coukos* reported on chemokine regulation of T cell responses in ovarian cancer. CCL28 is overexpressed in the hypoxic ovarian tumor microenvironment. Suppressive Tregs are recruited and accumulate at the tumor site through CCR10 and CCL28. In mice, anti–CCR10-ZAP (immunotoxin) is an effective tool to deplete non specific Tregs, without affecting CD8 T cells that instead express CCR3. Interestingly, CCL28 over-expression also correlated with enhanced VEGF expression; thus highlighting the synergy between immune tolerance and angiogenesis, as repair mechanisms adopted by the tumor. *M. Guidoboni*, MD (IRST Cancer Center Italy) discussed how DC vaccination may concurrently reduce Tregs and enhance activated CTLs in tumor biopsies from immunoresponsive patients with advanced melanoma. Pre- and post-vaccination TILs showed increased proportions of CD8 T cells without accumulation of Tregs. In progressing patients, post-vaccination lesions had decreased specific tumor-antigen expression, suggesting selection of tumor cells expressing different antigens. *W. H. Fridman* discussed how tumor infiltration by various subsets of immune cells can impact prognosis in analyses of large cohorts of primary colorectal carcinoma. An immune score based on tumor infiltration by T cells seems to be a better prognostic factor in colorectal cancer compared with the classical TNM staging system. A meta-analysis showed a >90% concordance between presence of Th1 (CD8+ CD45RO+) cells and good prognosis; while, presence of Th17 cells seem to be associated with poor prognosis. In lung cancer, surrounding high endothelial venules that promoted B and T cells trafficking, lead to formation of tertiary lymphoid structures (tumor-induced bronchus-associated lymphoid tissues) that were associated with favorable outcome. *S. Adams*, MD (New York University Cancer Institute) showed how topical TLR7 agonist, imiquimod, can induce immune–mediated rejection of breast cancer skin metastases. Imiquimod applied on skin metastases showed similar safety than in genital warts and led to improved clinical outcome especially after subsequent therapy. *A. Wesa*, PhD (Celsense Inc., Pittsburgh) reported on serial imaging of inflammation and therapeutic response with 19F MRI. In rat models of rheumatoid arthritis, 19 F imaging visualized phagocytic infiltration in the limb, which correlated with disease status. *V. Bronte*, MD (Immunology Dpt., University of Verona, Italy) discussed how post-translational chemokine modification can prevent intratumoral infiltration of antigen-specific T cells. Thus, in addition to directly causing TIL unresponsiveness to stimuli, reactive nitrogen species generated in the tumors can induce a permanent modification by nitration of CCL2 (N-CCL2). N-CCL2 cannot attract TIL to the core of the tumor, while recruitment of monocytes expressing higher levels of CCR2, is retained. The use of a novel nytrilation inhibitor (AT-38) in a murine model of prostate cancer led to decreased N-CCL2 and stronger T cell infiltration. In an adoptive therapy model, AT-38 changed the tumor microenvironment leading to better CTL chemotaxis and tumor regression.

## Prostate cancer as a learning model

This session was co-chaired by *C. Drake*, MD, PhD (John Hopkins University) and *J. Gulley*, MD, PhD (National Cancer Institute). *C. Drake* presented results on immune checkpoint blockade in prostate and other cancers. A single course of PD-1 blockade (MDX106) administered two months after vaccination (GVAX) could lead to tumor regression in a phase I trial in prostate cancer. Immunohistological examination of tissue microarrays of prostate tissue (from normal, benign hypertrophy, and carcinoma samples) showed rather constant Treg or Treg/CD8 ratio, while CD8/CD4 infiltration was higher in normal versus malignant tissues. Fresh lymphocytes from prostate gland needle biopsy (PIL) showed that 80% of CD8 T cells expressed PD-1 and were functionally inactive. PD-1 blockade was able to reverse proliferative defect of PIL if Gleason score was = 6 but not if > 7. In a murine model of glioblastoma, radiation combined with PD-1 blockade resulted in higher tumor regression. *D. McNeel*, MD, PhD (University of Wisconsin) discussed how repeated DNA vaccination can elicits prostatic acid phosphatase (PAP) antigen–specific T cell immune responses in patients with castration-resistant prostate cancer. Cellular immune responses, but not antibodies, to this DNA vaccine were augmented after multiple injections, though some patients never responded. An increase in PAP-specific IFN-γ responses was associated with a favorable change in PSA doubling time. *K.-J. Kallen*, MD, PhD (CureVac, Germany) reported that intradermal immunization with a novel mRNA based vaccination technology induced strong T and B cell responses in Phase I/II a trials in non-small cell lung cancer and prostate carcinoma (PCA). CV9103 consisted of a mixture of modified and active mRNA coding for multiple prostate tumor antigens able to induce both humoral and cytolytic immune responses. After 5 vaccine doses, T cell responses were measured in 80% of PCA patients with about 60% responding to more than one antigen. *T. De Gruijl*, PhD (VU University Medical Center, Netherlands) discussed about lymphoid and myeloid biomarkers for clinical outcome of ipilimumab and prostate GVAX treatment and showed that tumor-related CTLA-4 expression by CD4+ T cells may be a dominant predictor of survival. Among 28 patients treated with GVAX and anti-CTLA-4, 5 showed partial regression and 12 stable disease. Occurrence of autoimmune adverse events was associated with clinical response. Immunomonitoring showed that increase in ICOS + FoxP3+ Tregs and MDSCs was associated with shorter survival, while high pretreatment frequencies of CD4 + CTLA4+ (but not Tregs) correlated with longer survival. Cluster analysis correlated low activation status with poor survival, and high DC/low MDSC/low Treg pattern with good survival. *M. Provenzano*, PhD (University of Zurich, Swizterland) presented a comprehensive characterization of polyomavirus BK large tumor antigen (L-Tag) epitopes that promote the expansion of effector T lymphocytes in prostate cancer patients. Four potentially immunogenic BK L-Tag peptides were identified with a strong ability to reactivate and maintain CTL in prostate cancer patients. *J. Gulley* concluded the session by presenting how combining vaccines with other therapeutics may be a strategy to accelerate proof-of -concept studies. In contrast to chemotherapy, vaccination does not improve time to progression (TTP), but increases overall survival. This is due to profound differences in the mechanism of action of chemotherapy and immunotherapy. While chemotherapy rapidly, yet transiently, kills sensitive tumor cells, immunotherapy, by boosting endogenous immunity, can support cytotoxic and long-lasting anti-tumor responses. Combination of targeted radiation (Quadramet) or androgen deprivation (Flutamide) with PSA-TRICOM vaccine showed longer TTP than the single therapy. Similarly, combining a vaccine (PANVAC) with Docetaxel in breast cancer patients prolonged TTP to 274 days, as compared with 182 days with Docetaxel alone.

## Genetically engineered receptors and adoptive cell therapies

Researchers have long tried to harness the power of the immune system to fight cancer and overcome immune tolerance towards cancer cells; however, defining how to best target functional T cells remains challenging. To date, tumor immunotherapy with T cells, which can recognize and destroy malignant cells, has been limited by the ability to isolate and expand tumor-antigen specific T cells from the tumor (TIL). Gene transfer technologies provide the means to genetically modify T cells to stably express antibody binding domains on their surface that confer novel antigen specificities irrespective of HLA type. *C. June* and his collaborators, Penn University, Philadelphia, have genetically modified T cells to express a chimeric antigen receptor (CAR) to yield CAR^+^ T cells expressing an anti-CD19 CAR including both CD3-ζ and the 4-1BB costimulatory domain (CART19 cells) to target chronic lymphocytic leukemia (CLL), the second most common type of leukemia in adults affecting B cells. In a pilot clinical trial of CART19 cell adoptive transfer into three CLL patients, these cells expanded > 1000-fold, persisted more that 6 months in vivo and, retained anti-leukemic effects in a CD19-specific fashion in all three patients examined. On average, each infused CAR^+^ T cell was estimated to kill at least 1000 CLL cells. Some evidence for on-target toxicity included B cell aplasia and hypogammaglobulinemia. It was also found that those CART19 cells persisted as both central and effector memory T cells, which likely explains their longevity in vivo. The impressive results achieved with this cellular therapy highlight the potential of CAR-engineered T cells to usher in a new chapter in cancer therapy; however, they generate some compelling questions: why were CART19 cells not rejected? What is the mechanism of long-term persistence, is it antigen-dependent or independent? And finally, what is the long-term safety of CAR-engineered T cells?

In line with recent developments to improve adoptive T cell therapy of cancer, *D. Schendel,* Helmholtz Center, Munich, and her collaborators described unanticipated drawbacks in the application of allorestricted peptide-specific T cells as sources of high-affinity TCR. As a target antigen for adoptive T cell therapy, they selected survivin, an apoptosis inhibitor protein that is overexpressed in many tumors. To obtain lymphocytes expressing high affinity survivin-specific T cell receptors, they isolated HLA-A2–allorestricted survivin-specific T cells with high functional avidity. HLA-A2^+^ but not HLA-A2^–^ survin-expressing lymphocytes underwent extensive apoptosis due to HLA-A2–restricted fratricide of survivin-expressing lymphocytes, which naturally express ligands for specific TCR recognition. These results raise a general question regarding the development of cancer vaccines that target universal antigens (i.e.: survivin and telomerase) expressed by a variety of host cell types, including activated lymphocytes. Induction of high-avidity T cells might, indeed, limit themselves by self-MHC–restricted fratricide and elimination of neighboring T cells of other specificities.

## Session: high throughput technologies for immune monitoring

With the rapid advances in the development of potent and specific immunomodulators as well as personalized cellular therapies for control of cancer progression, the design and validation of high throughput technologies for immune monitoring represent a very hot topic among immunologists and oncologists. These would ideally allow the identification of biomarkers and baseline patient characteristics associated with a response to a given therapeutic approach and would ultimately translate into the design of algorithms that guide clinical decision.

At the SITC Annual meeting, a whole session was dedicated to this topic and several outstanding speakers lead the discussion. In the specific, *S. Gnjatic,* Ludwig Institute for Cancer Research at Memorial Sloan Kettering Institute, New York, shared the great progress made in the definition of humoral as well as cellular biomarkers associated with response to treatment with Ipilimumab, a monoclonal antibody against cytotoxic T lymphocyte antigen 4 (CTLA-4), which has been shown to improve survival in patients with advanced metastatic melanoma. It also enhances both cellular and humoral immunity to NY-ESO-1, a cancer/testis antigen expressed in a subset of patients with melanoma. NY-ESO-1-seropositive patients, either at baseline or post-treatment, had a greater likelihood of experiencing clinical benefit 24 wk after ipilimumab treatment than NY-ESO-1-seronegative patients (P = 0.02, relative risk = 1.8, two-tailed Fisher test). Interestingly, NY-ESO-1-seropositive patients with associated CD8^+^ T cells experienced more frequent clinical benefit (10 of 13; 77%) than those with undetectable CD8^+^ T cell response, as well as a significant survival advantage (P = 0.01; hazard ratio = 0.2, time-dependent Cox model). Serological analysis of arrays displaying the complete human proteome (seromics) along with multiparametric T cell assays represent a new era in cancer immunology and support their validation in prospective studies.

Another yet-in-progress battle against cancer lies in the identification of novel antigenic targets for immunotherapy. In this regard, *P. Beckhove*, German Cancer Research Institute, Heidelberg, presented the results generated with the ProteomeLab PF2D technology, in which TAA analysis can be tailored to an individual patient. The investigators applied this method to human tissue derived from head and neck as well as brain tumors and identified MUC1 and EGFR as tumor-associated antigens selectively recognized by T cells in H&N cancer patients. In addition, they identified CD4^+^ and CD8^+^ T cell responses against two novel antigens transthyretin and calgranulin B/S100A9a in a patient with a malignant brain tumor. This is emerging as a fast and inexpensive method to identifying novel and potentially immunogenic TAA irrespective of cell type and HLA haplotype.

## State of the art of animal models and veterinary applications for cancer and immunology

*T. Blankenstein*, PhD, Max-Delbruck Center for Molecular Medicine, and *J. D. Wolchok*, MD, PhD, Memorial Sloan-Kettering Cancer Center, co-chaired a session on developing advances in animal models for cancer and immunology. *T. Blankenstein* presented results of tumor immune escape in a mouse model of sporadic cancer. Conventional therapy results in initial tumor regression, which leads to subsequent growth of resistant cell clones. In this mouse model, tumors originating from drug-resistant clones can be eradicated by adoptive transfer of a specific CD8+ effector T cell. He demonstrated that adoptively transferred T cells, as opposed to chemotherapy, modulate tumor vasculature promoting bystander elimination of tumor cells.

*S. Spranger*, Helmholtz Zentrum München, Germany, discussed a mouse model that allows testing of human dendritric cell-based immunotherapies and comparisons in vivo of different vaccine strategies. Using an NOD/scid/IL2Rγnull (NSG) mouse model, she observed that vaccination using human-derived DCs matured with a cocktail containing TLR7/8 agonists resulted in enhanced immune responses. This model also allows testing of different DC variants, as well as the immunogenicity of different immunizing antigens.

*W. J. Murphy*, PhD, University of California-Davis, developed a xenogeneic model of orthotopic glioblastoma multiforme (GBM). He tested intracranial transfer of human NK cells in combination with a human IL-15 gene therapy in NSG mice grafted with a GBM cell line. Murphy observed an increased NK cell engraftment due to the secretion of IL-15 and a significant tumor volume reduction., This therapy did not protect mice from long-term tumor relapse and death, however, it demonstrated the feasibility of NK cell-based therapies for gliomas.

*T. Merghoub*, PhD, Memorial Sloan-Kettering Cancer Center, presented transgenic mouse models of spontaneous melanoma created by expressing oncogenes implicated in tumor development. TG-3 and Grm1 transgenic mouse strains develop melanoma at different stages of tumor progression. Merghoub showed that immunization with an optimized TYRP1 DNA vaccine, while preventing Grm1 melanoma progression, resulted inefficient in the aggressive TG-3 mouse model. He demonstrated that resistance to treatment is due to higher levels of regulatory T cells, and emphasized the importance of combining vaccines with immune modulators.

## Presidential abstract session

The session brought together young investigators who received the SITC presidential travel awards. All four scientists were selected for 20-minute oral presentations. This session was chaired by *T. F. Gajewski*, MD, PhD, University of Chicago, the current SITC president.

*J. Brody*, MD, Stanford University Medical Center, presented the development of “immunotransplant” combining CpG vaccination, vaccine-primed T cell harvest, myeloablation with stem cell rescue, and T-cell re-infusion. In a preclinical model of lymphoma, immunotransplant induces a preferential Teff:Treg ratio and amplifies anti-tumor T cells leading to the cure of systemic lymphoma. In patients with mantle cell lymphoma, immunotransplant increases the proportion of tumor-reactive T cells in 83% of patients and the upregulation of activation markers.

*L. Uccellini*, PhD, Department of Transfusion Medicine, NIH, presented an innovative study which analyzed associations between melanoma immune responsiveness and IRF5 polymorphism in TILs. His results support that polymorphism of IRF5 appears to be a predictor of the immune response to adoptive therapy with TILs in metastatic melanoma.

*L. V. Hurton*, MD Anderson Cancer Center, The University of Texas-Houston, discussed the improvement of transferred T-cell persistence in vivo. Hurton highlighted the necessity to find alternatives to systemic administration of IL-2 to stimulate T-cell expansion because of its high toxicity. She generated a membrane-bound IL-15 molecule (mbIL-15) to deliver localized IL-15 signaling to T-cells. Human T cells were genetically modified to express mbIL-15 in combination with CD19-specific CAR. In mice bearing CD19+ malignancies, adoptively transferred mbIL-15 + CAR + T cells demonstrated a better in vivo T cell persistence and anti-tumor effect, without the administration of exogenous cytokines.

*Y. Zheng*, PhD, Pathology, University of Chicago, focused on the functional anergy of TILs. She reported that early growth response 2 transcription factor (Erg2) is a central regulator of T cell anergy. Deletion of Egr2 prevents anergy *in vitro* and *in vivo*. This transcription factor directly regulates most of the known anergy-related genes like Crtam and Lag3. In the B16 melanoma mouse model, she showed that 40–60% of TILs that upregulated Crtam and Lag3 represented a subpopulation of PD-1+ T cells with defective IL-2 production. She concluded that cell surface markers to identify anergic T cells might be useful to define therapeutic targets for immunotherapy.

## Competing interests

The authors declare that they have no competing interests.

## Authors’ contributions

All authors contributed to writing of these highlights and all read and approved the final manuscript.

